# Macrophage pyroptosis in lupus nephritis – Novel insights into renal pathogenesis and clinical correlates: A cross-sectional study

**DOI:** 10.1097/MD.0000000000050930

**Published:** 2026-09-25

**Authors:** Kun Ye, Qian Yang, Tingting Jiang, Runhang Chen, Yunfeng Huang, Yiyun Huang, Jiao Lan, Lingzhang Meng

**Affiliations:** a Guangxi Clinical Research Center for Chronic Kidney Disease & Department of Nephrology, Guangxi Academy of Medical Sciences & The People’s Hospital of Guangxi Zhuang Autonomous Region, Nanning, China; b Department of Nephrology, Guangxi Hospital Division of The First Affiliated Hospital, Sun Yat-sen University, Nanning, China; c Research Center of Medical Sciences, Guangxi Academy of Medical Sciences & The People’s Hospital of Guangxi Zhuang Autonomous Region, Nanning, China; d Research Center for Medical Big Data and Artificial Intelligence, Guangxi Academy of Medical Sciences & The People’s Hospital of Guangxi Zhuang Autonomous Region, Nanning, China.

**Keywords:** clinical features, lupus nephritis, macrophage pyroptosis

## Abstract

Pyroptosis is a form of programmed cell inflammatory death. Macrophage pyroptosis has been implicated in the development of various immune diseases, yet its role in lupus nephritis (LN) remains poorly understood. This study examined macrophage pyroptosis in LN and its clinical correlations. This was a cross-sectional observational study. Renal tissues and clinical data were collected from 49 LN patients and 20 minimal change disease (MCD) patients. Macrophage pyroptosis was detected using immunofluorescence (cluster of differentiation 68 [CD68]/gasdermin D [GSDMD] co-staining), and serum inflammatory markers were analyzed via ELISA. Hierarchical regression analyses were performed to identify clinical parameters significantly associated with macrophage pyroptosis. A comparative analysis between the LN cohort (41 females, 8 males; mean age 32.6 ± 12.1 years) and MCD controls revealed significantly elevated levels of blood urea nitrogen, C-reactive protein, uric acid, and anti-double-stranded DNA antibodies (*P* < .05), accompanied by decreased complement component 3 (C3), complement component 4 (C4), estimated glomerular filtration rate (eGFR), and total cholesterol. Immunofluorescence demonstrated a marked increase in CD68/Cleaved GSDMD double-positive cells within both glomerular and interstitial compartments of LN patients (*P* < .001). LN patients with chronic kidney disease (CKD) stages 5 exhibited significantly interstitial macrophage pyroptosis (*P* < .05). This study is the first to report the presence of macrophage pyroptosis in LN renal tissues, with the extent of interstitial pyroptosis showing a significant cross-sectional association with CKD stage (by eGFR). This observation suggests a potential link between macrophage pyroptosis and disease severity in LN, meriting further investigation.

## 1. Introduction

Systemic lupus erythematosus (SLE) is an autoimmune disorder that affects multiple systems and organs in the body, including the central nervous system, heart, skin, and kidneys.^[1]^ Kidney damage associated with SLE is known as lupus nephritis (LN). Numerous studies have indicated that adolescent – onset SLE patients have a higher prevalence of LN (50–82%) compared to adult – onset SLE patients (34–53%).^[2]^ As a result, LN is a major contributor to morbidity and mortality in SLE patients.^[3]^ Although treatments such as hormones, immunosuppressants, biological agents, and plasma exchange can alleviate the symptoms of LN, they are accompanied by serious side effects, including bone marrow suppression, severe infections, and liver and kidney damage. Due to the disadvantages of poor and easily recurrent outcomes, unclear long-term efficacy, and a substantial economic burden, up to a quarter of LN patients progress to end-stage renal disease (ESRD).^[4]^ Therefore, an improved understanding of the pathogenic mechanisms underlying LN may provide insights that could inform the development of novel and effective treatments.

A series of studies has focused on the pathogenesis of LN, investigating inflammatory kidney damage induced by factors such as the deposition of immune complexes, complement activation, and infiltration of immune cells.^[5,6]^ Notably, in most immune-associated nephritis cases, macrophages are a significant infiltrating cell population in renal tissue, and they are closely linked to the severity of proteinuria, renal function decline, crescent formation, and interstitial fibrosis.^[7]^ When macrophages infiltrate the kidney, they can intensify the inflammatory response through antigen presentation or involvement in tertiary lymphoid tissue. Additionally, infiltrated and resident macrophages in the kidney may continuously contribute to renal damage.^[8]^ Given the abundant presence of macrophages and obvious inflammation in the renal tissue of LN, it is relevant to explore whether macrophage-associated inflammatory responses play a role in the occurrence and development of LN.

Pyroptosis, first identified in macrophages of mice infected with Salmonella typhi in 1992 by Zychlinsky and later confirmed to be mediated by the Gasdermin family, is a mode of cell death.^[9,10]^ In contrast to apoptosis, pyroptosis occurs more rapidly, with pyroptotic cells characterized by swelling, rupture, and the release of inflammatory cell contents.^[11]^ Thus, pyroptosis is essentially a pro-inflammatory process. In the classical pyroptosis pathway, upon receiving stimulatory signals, cells assemble inflammasomes that activate caspase-1. Activated caspase-1 then cleaves Gasdermin-D (GSDMD), leading to the formation of plasma membrane pores and the release of cellular contents. This process can promote the maturation and release of interleukin-18 (IL-18) and interleukin-1β (IL-1β), thereby recruiting more inflammatory cells and further amplifying the inflammatory response.^[11,12]^ Numerous studies have focused on elucidating the pathogenic significance of pyroptosis in SLE. Reports have indicated the presence of pyroptosis in the peripheral blood or kidney tissue of SLE/LN patients and animal models. In this context, pivotal pyroptosis-associated molecules, including NLRP3 (NOD-like receptor family pyrin domain containing 3), AIM2 (absent in melanoma 2), caspase-1, HMGB-1 (high mobility group box 1 protein), IL-18, and IL-1β, are highly expressed and show a positive correlation with anti-double-stranded DNA (anti-dsDNA) levels and disease severity.^[13]^ This strongly suggests that pyroptosis may drive the progression of LN. However, the specific mechanism remains to be fully clarified. Pyroptosis is mainly found in monocytes, macrophages, and dendritic cells, representing a distinct inflammatory response mechanism.^[14]^ While macrophage pyroptosis has been extensively studied in conditions such as acute lung injury, knee osteoarthritis, sepsis and ulcerative colitis,^[15-18]^ its role in LN progression remains unclear.

Therefore, our study aimed to investigate the presence of macrophage pyroptosis in renal tissue of LN patients and explore the correlation between the degree of macrophage pyroptosis and clinical indicators. We anticipate that our findings will provide new insights into the mechanisms underlying the occurrence and development of LN.

## 2. Materials and methods

This study was conducted in accordance with the Strengthening the Reporting of Observational Studies in Epidemiology (STROBE) guidelines for cross-sectional studies.

### 2.1. Study subjects

A total of 49 LN patients and 20 MCD patients, diagnosed by renal biopsy in the Department of Nephrology at the People’s Hospital of Guangxi Zhuang Autonomous Region between January 2019 and January 2021, were included in this study. The inclusion criteria for LN patients were as follows: fulfillment of the 1997 American College of Rheumatology (ACR) revised diagnostic criteria for SLE; first-time renal biopsy confirmation of LN after SLE diagnosis; and availability of complete research data. The exclusion criteria included renal pathology showing non-LN histopathological changes; concurrent immune-related disorders (including rheumatoid arthritis, Sjögren’s syndrome, or Henoch-Schönlein purpura); and comorbidities of malignancies, tuberculosis, hepatitis B, or severe infections. For MCD patients, the inclusion criteria were meeting the Kidney Disease: Improving Global Outcomes (KDIGO) diagnostic criteria for MCD and having complete research data., The exclusion criteria were coexisting immune-related diseases, malignant tumors, tuberculosis, hepatitis B, severe infections, or renal pathology exhibiting non-MCD histopathological alterations. The exclusive recruitment of participants from a single regional medical center introduces potential selection bias, which may limit the generalizability of our conclusions to broader populations, a limitation acknowledged under STROBE guidelines for single-center studies.

The project was approved by the Medical Ethics Committee of the People’s Hospital of Guangxi Zhuang Autonomous Region (Approval#: KY-GZR-2022-074). All patients provided written informed consent for their renal biopsy and the use of their clinical data for medical purposes at the time of admission. Given the retrospective nature of this observational study and the use of anonymized data for secondary analysis, the requirement for additional informed consent was waived by the Ethics Committee. Kidney tissues used in this study were residual specimens obtained from routine renal biopsies performed between January 2019 and January 2021. Due to ethical and practical limitations, healthy renal controls could not be used. MCD tissues were selected as controls because they are characterized by low inflammatory infiltration, making them a feasible comparator for pyroptosis analysis. Patients were not involved in the design, conduct, reporting, or dissemination plans of this research.

### 2.2. Data collection

Clinical symptoms and laboratory results were collected retrospectively. Information was gathered from 3 aspects: General information, including age, sex, course of disease (COD), and treatment history, with a particular focus on the use of immunosuppressive agents (ISA); laboratory examination results, including proteinuria, renal function tests, liver function tests, blood lipids, serum complement (C3, C4), autoantibody tests, and the Systemic Lupus Erythematosus Disease Activity Index (SLEDAI); and Activity Index (AI) and Chronicity Index (CI) scores from renal pathology. Two doctors served as data collectors, and all patient information was kept confidential.

### 2.3. Experimental antibodies

CD68 is a marker for macrophages, and GSDMD is a key molecule in pyroptosis. Double immunofluorescence staining of CD68 and Cleaved GSDMD was performed on kidney tissue sections from patients to assess the level of macrophage pyroptosis. The details of the antibodies used are as follows: The CD68 antibody was procured from Nakasugi Golden Bridge (#ZM-0060, China), and the Cleaved GSDMD antibody was obtained from Cell Signaling Technology (#36425S, USA). The secondary antibodies, goat anti-rabbit IgG (#ab150084) and goat anti-mouse IgG (#ab150117), were purchased from Abcam (UK).

### 2.4. Double immunofluorescence

First, sections were dewaxed, rehydrated, and subjected to ethylenediaminetetraacetic acid (EDTA) repair for 10 minutes, followed by cooling to room temperature. Then, the slides were immersed in 5% bovine serum albumin (Solarbio, China) to block non-specific binding for 20 minutes at room temperature. Sequentially, the slides were incubated with the primary antibodies CD68 (1:100) and Cleaved GSDMD (1:200) overnight at 4°C in the dark. After washing, the slides were treated with anti-mouse and anti-rabbit secondary antibodies (1:200) at 37°C for 120 minutes in the dark. Subsequently, the slides were incubated with 4’,6-diamidino-2-phenylindole (DAPI; Bioss, China) for 10 minutes at 37°C, protected from light. Finally, the slides were mounted using an anti-fluorescence quencher (Melan Bio). The results were observed and photographed under a fluorescence microscope. To confirm staining specificity, negative controls were systematically processed by omitting the primary antibodies and incubating the sections with blocking buffer alone, followed by secondary antibody incubation. No significant nonspecific fluorescence or background signal was observed in these control sections.

For each patient’s renal tissue, all glomeruli within each section were imaged, and 10 randomly selected, non-overlapping fields of the renal interstitium were captured using a microscope with a 400×  magnification. To ensure unbiased selection, interstitial fields were chosen systematically by moving the stage in a predetermined pattern. while avoiding glomeruli and large vessels. CD68-positive cells (single-positive) and CD68/Cleaved GSDMD double-positive cells were manually quantified using the Cell Counter plugin in ImageJ. All quantifications were performed by 2 independent observers who were blinded to the clinical data. To evaluate the reliability of the quantification, the quantitative inter-observer agreement was assessed using the Intraclass Correlation Coefficient (ICC), yielding an ICC value of 0.89, indicating excellent reproducibility. The number of positive cells per glomerulus was averaged across all glomeruli in each section to obtain the mean number of positive cells per glomerulus for each patient. Inter-observer variability was assessed, and any discrepancies prior to final averaging were resolved by consensus or by consultation with a third observer.

### 2.5. Enzyme-linked immunosorbent assay

The levels of IL-1β and IL-18 in patients’ serum were measured using ELISA kits (Fankew, Shanghai FANKEL Industrial Co, Ltd, China) according to the manufacturer’s instructions.

### 2.6. Statistical analysis

No formal power calculation was conducted prior to the study; the sample size was based on the availability of eligible patients with complete biopsy and clinical data during the study period (January 2019 to January 2021). Data completeness was verified for all enrolled participants. Cases with missing key variables (e.g., incomplete laboratory results or insufficient tissue for immunofluorescence) were excluded prior to analysis. No imputation methods were used for missing data as the final cohort consisted only of patients with complete datasets.

The data were statistically analyzed using SPSS 27.0 software (International Business Machines Corporation). For measurement data conforming to a normal distribution, descriptive statistics such as mean ± standard deviation were used. The comparison between 2 groups was performed using the independent samples *t* test. For data not conforming to a normal distribution, descriptive analysis was conducted using the median (interquartile range), and the comparison between 2 groups was carried out using the Mann-Whitney *U* test. Count data were presented as percentages, and the chi-square test was used for between-group comparisons. Given the multiple parameters evaluated, the preliminary Correlations were evaluated using Spearman correlation analysis. These univariate analyses were exploratory in nature, and because no formal multiplicity correction was applied, the unadjusted $P$-values should be interpreted cautiously. All tests were 2-tailed, with a significance level set at *P* < .05. To further explore the potential associations between clinical parameters and macrophage pyroptosis, while adjusting for potential confounders, hierarchical multiple regression analysis was performed.

## 3. Result

### 3.1. Demographic and clinical data

According to the inclusion/exclusion criteria, 69 patients were enrolled in this study (49 with LN and 20 with MCD). The MCD group had a 1:1 male-to-female ratio, while LN patients were predominantly female (41 females, 83.7%), showing a statistically significant gender difference (*P* = .004). The disease duration in the LN group was significantly longer than in the MCD group (*P* = .02). Notably, 23 LN patients (46.9%) had received immunosuppressive therapy, compared to only 1 MCD patient (5%). The LN group had poorer renal function than the MCD group, as evidenced by a lower estimated glomerular filtration rate (eGFR; *P* < .001). Inflammatory markers such as erythrocyte sedimentation rate (ESR) and C-reactive protein (CRP) were significantly higher in the LN group (*P* < .05). LN-specific markers (C3, C4, and anti-dsDNA) were also significantly different between the LN group and the MCD group (*P* < .001). The total cholesterol (TC) level in the MCD group was higher than that in the LN group (*P* = .016). No significant differences were observed in albumin (Alb), serum creatine (SCr), 24h Urine protein (24h UPro), triglyceride (TG), or Vitamin D (VitD) levels between the 2 groups (Table 1).

**Table 1:** Comparison of clinical data of patients with LN and MCD.

Parameters	LN	MCD	*t/Z/χ^2^*	*P* value	95% CI
Number	49	20			
Gender, n (%)			8.352	.004	(0.06, 0.62)
Male	8 (16.3)	10 (50)			
Female	41 (83.7)	10 (50)			
Age (yr)	32.6 ± 12.1	29 (16–49)	−0.536	.592	(−7.00, 10.00)
COD (d)	90 (30–730)	30 (9–228)	−2.332	.020	(0.00, 172.00)
Treatment (ISA), n (%)			11.012	<.001	(2.08, 135.57)
Yes	23 (46.9)	1 (5.0)			
No	26 (53.1)	19 (95.0)			
Alb (45–55 g/L)	21.9 (20.0–28.4)	21.9 (17.7–35.5)	0.093	.926	(−5.20, 3.80)
BUN (2.6–7.5 mmol/L)	8.0 (5.4–12.0)	4.2 (3.5–5.6)	−3.293	<.001	(1.50, 5.49)
SCr (45–84 μmol/L)	83 (68–150)	77.4 ± 18.3	−1.687	.092	(−3.00, 43.00)
eGFR (≥60 mL/min/1.73 m^2^)	73.1 ± 33.9	110.6 ± 23.8	−5.208	<.001	(−51.93, −23.02)
UA (90–420 µmol/L)	483.1 ± 130.0	401.7 ± 124.7	2.390	.020	(13.41, 149.47)
24h UPro (0–0.15 g/24 h)	3.6 (2.1–5.3)	4.7 ± 3.4	0.417	.677	(−2.34, 1.64)
TC (<5.2 mmol/L)	5.3 (4.4–6.9)	10.4 ± 5.7	2.420	.016	(−7.41, −0.51)
TG (<1.7 mmol/L)	2.0 (1.6–3.0)	2.1 ± 1.3	-0.979	.328	(−0.33, 0.90)
Vit D (75–250 nmol/L)	26.1 (19.4–40.8)	17.1 (8.4–42.3)	−1.423	.155	(−3.35, 15.12)
ESR (0–20 mm/h)	72.0 ± 31.1	54.8 ± 23.2	2.234	.029	(1.84, 32.66)
C3 (0.79–1.52 g/L)	0.3 (0.2–0.5)	1.0 ± 0.2	6.231	<.001	(−0.71, −0.49)
C4 (0.16–0.38 g/L)	0.07 (0.04–0.12)	0.3 ± 0.1	5.544	<.001	(−0.20, −0.12)
Anti-dsDNA (≤0.5 IU/mL)	2.0 (0.5–3.9)	0.5 ± 0	−4.6	<.001	(0.37, 2.70)
CRP, n (%)			6.495	.011	(0.01, 0.81)
<5	32 (65.3)	19 (95.0)			
≥5	17 (34.7)	1 (5.0)			
SLEDAI, n (%)			–	–	–
0–4	4 (8.2)	–			
5–9	7 (14.3)	–			
10–14	18 (36.7)	–			
≥15	20 (40.8)	–			
AI, n (%)			–	–	–
≤8	35 (71.4)	–			
>8	14 (28.6)	–			
CI, n (%)			–	–	–
0	17 (34.7)	–			
1	12 (24.5)	–			
2	7 (14.3)	–			
3	6 (12.2)	–			
4	1 (2.0)	–			
5	4 (8.2)	–			
7	2 (4.1)	–			
ANA, n (%)		–	–	–	–
Negative	2 (4.1)	–			
<1: 640	16 (32.6)	–			
≥1: 640	31 (63.3)				
Pathological classification, n (%)		–	–	–	–
II	3 (6.1)	–			
III	2 (4.1)	–			
IV	10 (20.4)	–			
V	5 (10.2)	–			
III+IV	1 (2.0)	–			
III+V	4 (8.2)	–			
IV+V	24 (49.0)	–			

24h UPro = 24h Urine protein, 95% CI = 95% confidence intervals, a-dsDNA = anti-double-stranded DNA, AI = activity index, Alb = albumin, ANA = antinuclear antibodies, BUN = blood urea nitrogen, C3 = complement 3, C4 = complement 4, CI = chronic index, COD = course of disease, CRP = C-reactive protein, eGFR = estimated glomerular filtration rate, ESR = erythrocyte sedimentation rate, ISA = immunosuppressive agent, LN = lupus nephritis, SCr = serum creatine, SLE = systemic lupus erythematosus, SLEDAI = systemic lupus erythematosus disease activity index, TC = total cholesterol, TG = triglyceride, UA = uric acid, Vit D = vitamin D.

### 3.2. Comparison of macrophage pyroptosis levels between LN and MCD groups

LN patients showed prominent CD68-positive cells and CD68/Cleaved GSDMD double-positive cells in the glomeruli and interstitium, while only minimal CD68/Cleaved GSDMD double-positive cells were detected in MCD tissues (Figs. 1A and 2A). The difference between groups was statistically significant (*P* < .001, Figs. 1B, 2B, and Table 2). Further analysis revealed that serum IL-1β and IL-18 levels were significantly higher in the LN group than in the MCD group (*P* < .0001, Fig. 2C).

**Table 2:** Comparison of macrophage and macrophage pyroptosis in renal tissue of LN and MCD.

Parameters		LN	MCD	*Z*	*P* value	95% CI
Glomerulus	CD68/Cleaved GSDMD double-positive cells	1.08 (0.60–2.00)	0.04 (0–0.13)	−5.100	<.001	(0.70, 1.52)
	CD68 positive cells	5.44 (2.32–10.70)	0.48 (0.20–0.81)	−6.097	<.001	(3.74, 7.48)
Renal interstitium	CD68/Cleaved GSDMD double-positive cells	0.70 (0.20–1.50)	0 (0–0.05)	−4.719	<.001	(0.40, 1.10)
	CD68 positive cells	3.80 (1.40–6.10)	0.25 (0.02–0.65)	−5.091	<.001	(1.80, 4.70)

CD68 = cluster of differentiation 68 (macrophage marker), CI = Chronicity Index, GSDMD = gasdermin D, LN = lupus nephritis, MCD = minimal change disease.

**Figure 1.**
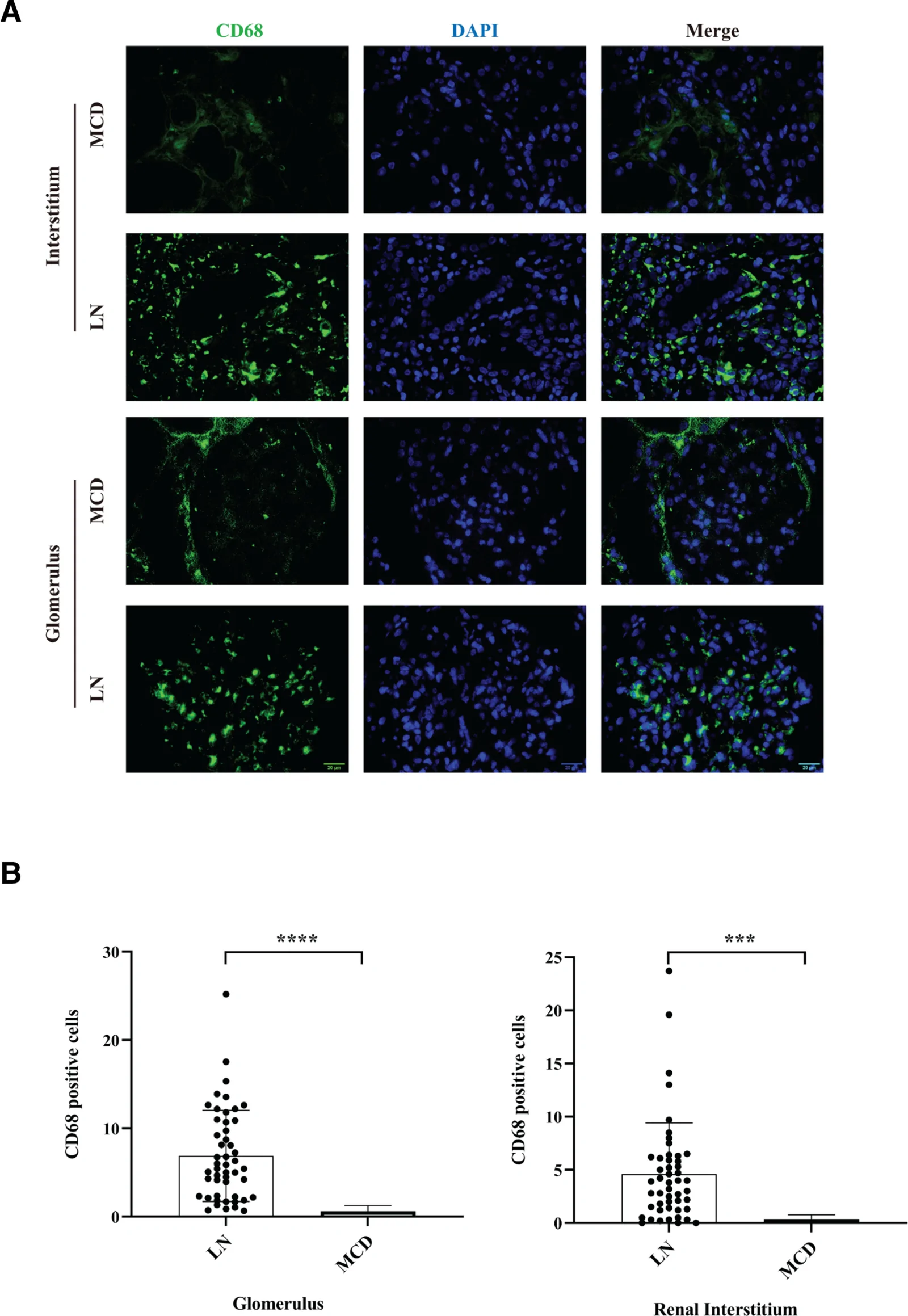
CD68 positive cells in renal tissue of LN and MCD patients. (A) LN had significant macrophage infiltration in glomerulus and interstitium as compared to MCD (green: CD68-positive cells; blue: DAPI nuclei). Scale bar: 20 μm. Immunofluorescence X400. (B) Differences between the 2 groups were statistically significant (*P *< .05), as determined by the Mann–Whitney *U* test. CD68 = cluster of differentiation 68 (macrophage marker), CI = Chronicity Index, DAPI = 4’,6-diamidino-2-phenylindole, LN = lupus nephritis, MCD = minimal change disease.

**Figure 2.**
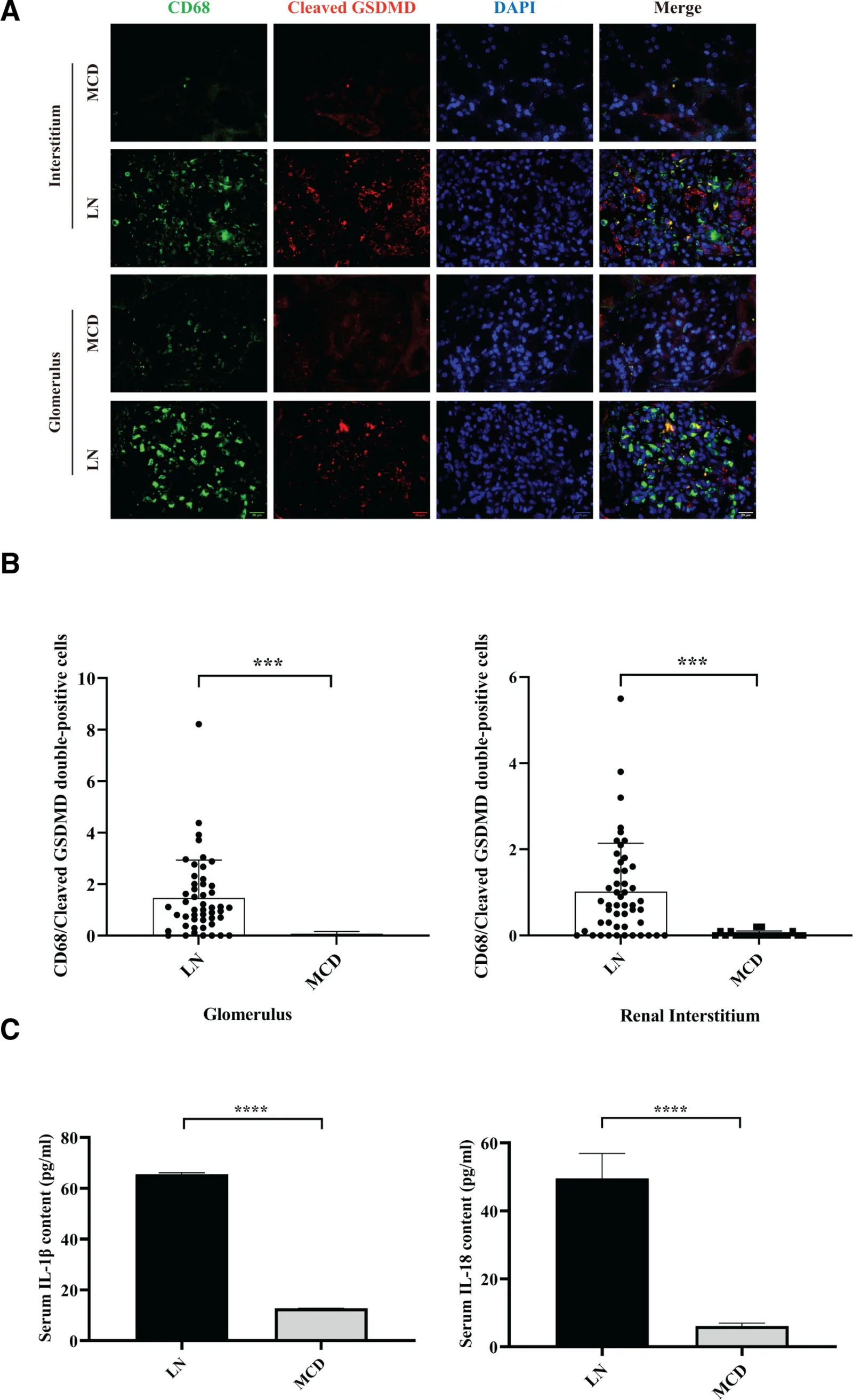
CD68/Cleaved GSDMD double-positive cells in renal tissue of LN and MCD patients. (A) Significant macrophage pyroptosis was shown in the LN compared to the MCD in the glomerulus and interstitium (green. CD68 positive cells; red. Cleaved GSDMD positive cells, yellow: CD68/Cleaved GSDMD double-positive cells; blue. DAPI nucleus). Scale bar: 20 μm. Immunofluorescence X400. (B) The differences between the 2 groups were statistically significant, *P* < .05. (C) Compared to MCD, LN serum showed considerably higher levels of IL-1β and IL-18. ***P < .001, ****P < .0001. Statistical analysis was performed using the Mann–Whitney *U* test. CD68 = cluster of differentiation 68 (macrophage marker), CI = Chronicity Index, DAPI = 4’,6-diamidino-2-phenylindole, GSDMD = gasdermin D, IL-18 = interleukin-18, IL-1β = interleukin-1β, LN = lupus nephritis, MCD = minimal change disease.

### 3.3. Correlation between the degree of macrophage pyroptosis in renal tissue and clinical data of LN patients

To investigate the relationship between macrophage pyroptosis and clinical parameters, Spearman correlation analysis was performed between LN indicators and pyroptosis levels for preliminary screening. It was found that glomerular macrophage pyroptosis was significantly correlated with uric acid (UA), ESR, C3, C4, and anti-dsDNA (*P* < .05, Fig. 3 and Table 3). Interstitial macrophage pyroptosis was also significantly correlated with blood urea nitrogen, SCr, eGFR, UA, TG, vitamin D (Vit D), ESR, CRP, and CI (*P* < .05, Fig. 3 and Table 3).

**Table 3:** Correlation between clinical indicators in LN patients and the level of macrophage pyroptosis in renal tissue.

Parameters	Glomerulus			Renal interstitium		
	*r*	*P*	95% CI	*r*	*P*	95% CI
Age	−0.024	.873	(−0.31, 0.27)	0.083	.573	(−0.21, 0.36)
COD	−0.110	.450	(−0.39, 0.19)	0.171	.241	(−0.13, 0.44)
Treatment (ISA)	0.120	.411	(−0.18, 0.40)	−0.239	.099	(−0.49, 0.05)
Alb	−0.040	.784	(−0.33, 0.25)	−0.086	.556	(−0.37, 0.21)
BUN	0.190	.191	(−0.11, 0.45)	0.319	.026	(0.03, 0.56)
SCr	0.245	.090	(−0.05, 0.50)	0.395	.005	(0.12, 0.61)
eGFR	−0.174	.233	(−0.44, 0.12)	−0.376	.008	(−0.60, 0.10)
UA	0.315	.028	(0.03, 0.55)	0.302	.035	(0.02, 0.54)
24h UPro	0.082	.575	(−0.21, 0.36)	0.279	.052	(−0.01, 0.53)
TC	−0.148	.310	(−0.42, 0.15)	0.030	.836	(−0.26, 0.32)
TG	0.005	.973	(−0.29, 0.29)	0.368	.009	(0.09, 0.59)
Vit D	−0.105	.474	(−0.38, 0.19)	−0.386	.006	(−0.61, −−0.11)
ESR	0.308	.032	(0.02, 0.55)	0.309	.031	(0.02, 0.55)
CRP	0.240	.097	(−0.05, 0.50)	0.412	.003	(0.14, 0.63)
C3	−0.320	.025	(−0.56, −0.03)	0.116	.428	(−0.18, 0.39)
C4	−0.455	.001	(−0.66, −0.19)	0.076	.602	(−0.22, 0.36)
a-dsDNA	0.310	.030	(0.02, 0.55)	−0.034	.819	(−0.32, 0.26)
ANA	0.171	.240	(−0.12, 0.44)	−0.108	.461	(−0.39, 0.19)
SLEDAI	0.067	.646	(−0.23, 0.35)	−0.160	.271	(−0.43, 0.14)
AI	0.000	1.000	(−0.29, 0.29)	0.008	.956	(−0.28, 0.30)
CI	0.170	.243	(−0.13, 0.44)	0.320	.025	(0.04, 0.56)

*r* represents the degree of correlation.

24h UPro = 24-hour urine protein, AI = Activity Index, AIM2 = absent in melanoma 2, Alb = albumin, ANA = antinuclear antibodies, anti-dsDNA = anti-double-stranded DNA antibodies, BUN = blood urea nitrogen, C3 = complement component 3, C4 = complement component 4, CD68 = cluster of differentiation 68 (macrophage marker), CI = Chronicity Index, COD = course of disease, eGFR = estimated glomerular filtration rate, ESR = erythrocyte sedimentation rate, ISA = immunosuppressive agents, LN = lupus nephritis, SCr = serum creatinine, SLEDAI = Systemic Lupus Erythematosus Disease Activity Index, TC = total cholesterol, TG = triglyceride, UA = uric acid.

**Figure 3.**
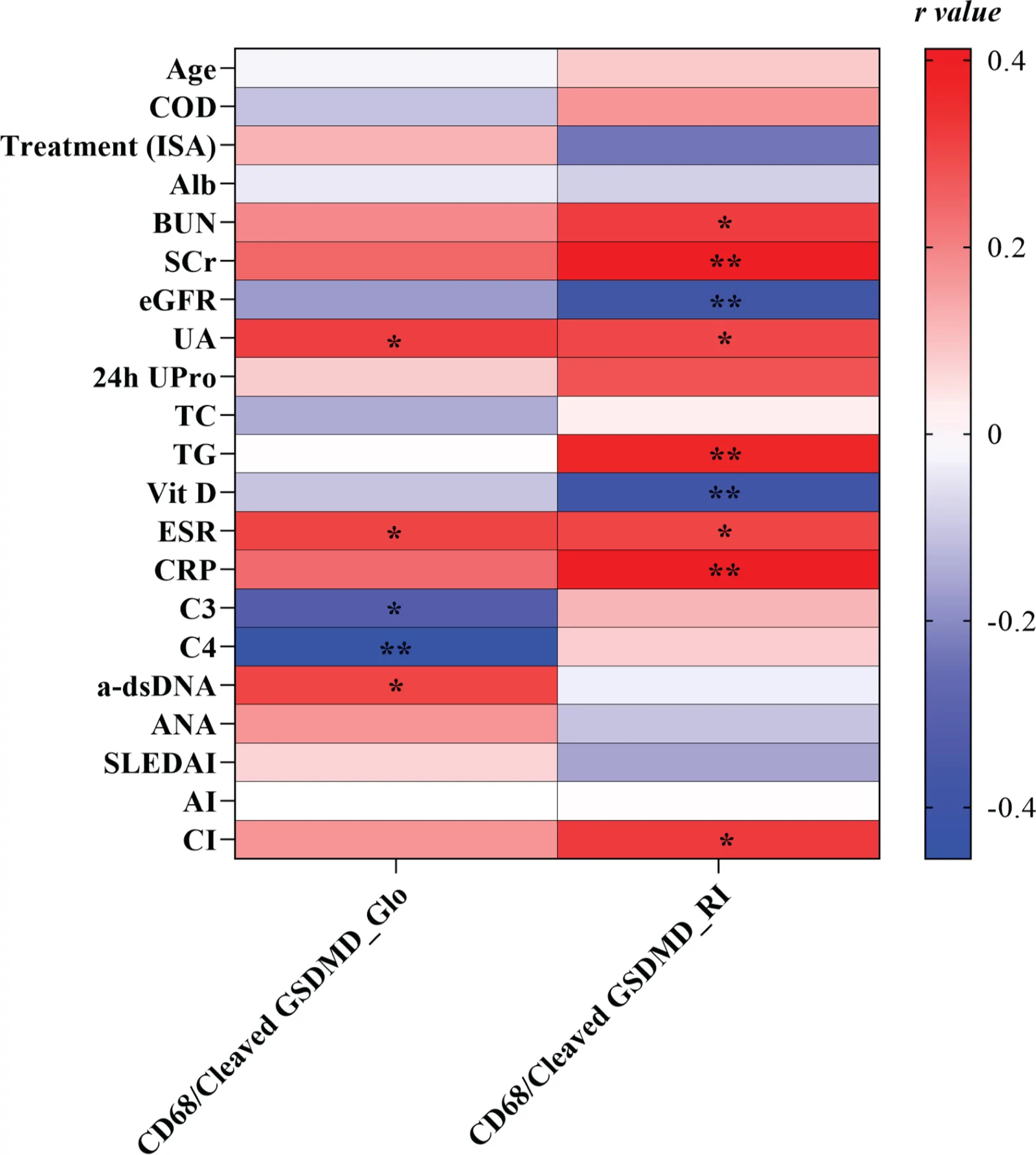
Visualization shows the relationship between macrophage pyroptosis and clinical indications. Color scale: red (positive correlation), blue (negative correlation); darker shades indicate stronger correlations. *P < .05, **P < .01. 24h UPro = 24h Urine protein, 95% CI = 95% confidence intervals, a-dsDNA = anti-double-stranded DNA, AI = activity index, Alb = albumin, ANA = antinuclear antibodies, BUN = blood urea nitrogen, C3 = complement 3, C4 = complement 4, CI = chronic index, CRP = C-reactive protein, eGFR = estimated glomerular filtration rate, ESR = erythrocyte sedimentation rate, GLO = glomerulus, ISA = immunosuppressive agent, LN = lupus nephritis, RI = renal interstitium, SCr = serum creatine, SLE = systemic lupus erythematosus, SLEDAI = systemic lupus erythematosus disease activity index, TC = total cholesterol, TG = triglyceride, UA = uric acid, Vit D = vitamin D. Statistical analysis was performed using Spearman correlation analysis.

Hierarchical multiple regression analyses were further conducted, adjusting for potential confounders (age, sex, disease duration, treatment history, and LN pathological class). When eGFR, UA, TG, VitD, ESR, and CRP were entered in Block 2, these variables collectively explained an additional 60.5% of the variance in interstitial macrophage pyroptosis (Δ*R*^2^ = 0.605, *P* < .001). In the final model, eGFR stage 5 showed the strongest independent association, accounting for the largest proportion of unique variance (β = 0.651, *P* < .001), followed by CRP (β = 0.352, *P* = .008) and VitD (β = −0.236, *P* = .039; Table 4).

**Table 4:** Hierarchical multiple regression analysis of clinical indicators on renal interstitium macrophage pyroptosis.

Parameters	*B*	SE	β	*t*	*R*	*R*2	Δ*R*2
Block 1					0.238	0.057	0.057
					*F*(5, 43) = 0.516		
COD	0.000	0.000	0.095	0.577			
Age	0.012	0.015	0.128	0.812			
Female	−0.293	0.458	−0.098	−0.639			
Male (ref.)							
Treatment (ISA)	0.188	0.392	0.085	0.481			
No (ref.)							
Class IV LN	0.153	0.371	0.062	0.412			
Others (ref.)							
Block 2					0.813	0.662	0.605***
					*F*(13, 35) = 5.263***		
COD	0.000	0.000	−0.002	−0.014			
Age	0.008	0.011	0.087	0.765			
Female	−0.230	0.353	−0.077	−0.653			
Male (ref.)							
Treatment (ISA)	0.529	0.299	0.239	1.770			
No (ref.)							
Class IV LN	−0.361	0.283	−0.147	−1.274			
Others (ref.)							
eGFR 3–4	0.244	0.337	0.108	0.726			
eGFR 5	5.103	0.917	0.651	5.565***			
eGFR1–2 (ref.)							
CRP ≥ 5	0.820	0.293	0.352	2.800**			
CRP < 5 (Ref.)							
VitD	−0.013	0.006	−0.236	−2.144*			
UA	0.000	0.001	0.029	0.203			
TG	−0.001	0.001	−0.152	−1.297			
ESR	0.003	0.005	0.093	0.708			
CI	−0.027	0.081	−0.045	−0.329			

Dependent variable was CD68/Cleaved GSDMD_RI. eGFR1–2 indicates CKD stages 1–2 as defined by eGFR criteria.

CD68 = cluster of differentiation 68 (macrophage marker), CI = Chronicity Index, CKD = chronic kidney disease, COD = course of disease, CRP = C-reactive protein, eGFR = estimated glomerular filtration rate, ESR = erythrocyte sedimentation rate, GSDMD = gasdermin D, ISA = immunosuppressive agents, LN = lupus nephritis, SCr = serum creatinine, SE = standard error, SLEDAI = Systemic Lupus Erythematosus Disease Activity Index, TC = total cholesterol, TG = triglyceride, UA = uric acid.

* *P* < .05.

** *P* < .01.

*** *P* < .001.

Since approximately 50% of the LN patients in this cohort had received immunosuppressive therapy, a subgroup analysis was performed to evaluate its potential impact. Statistical analysis demonstrated no significant difference in macrophage pyroptosis levels between immunosuppressant-treated and non-treated groups (*P* > .05). Notably, hierarchical multiple regression analysis further revealed that, among patients not receiving immunosuppressive therapy, advanced chronic kidney disease (CKD) stage 5 was associated with significantly higher levels of interstitial macrophage pyroptosis (β = 0.770, *P* = .002; Table 5).

**Table 5:** Hierarchical multiple regression analysis of clinical indicators on renal interstitium macrophage pyroptosis (immunosuppressant subgroups).

Parameters	*B*	SE	β	*t*	*R*	*R*2	Δ*R*2
Block 1					0.190	0.036	0.036
					*F*(4, 21) = 0.196		
COD	−0.000	0.000	−0.060	−0.279			
Age	0.003	0.025	0.029	0.118			
Female	0.295	1.025	0.064	0.288			
Male (ref.)							
Class IV LN	0.395	0.628	0.152	0.629			
Others (ref.)							
Block 2					0.871	0.758	0.722**
					*F*(12, 13) = 3.396*		
COD	0.000	0.000	0.054	0.346			
Age	0.002	0.020	0.020	0.098			
Female	0.210	1.204	0.045	0.174			
Male (ref.)							
Class IV LN	−0.125	0.581	−0.048	−0.216			
Others (ref.)							
eGFR 3–4	0.634	0.655	0.227	0.968			
eGFR 5	4.954	1.268	0.77	3.906**			
eGFR1–2 (ref.)							
CRP ≥ 5	0.662	0.502	0.260	1.319			
CRP < 5 (ref.)							
VitD	−0.010	0.011	−0.154	−0.931			
UA	0.000	0.002	−0.027	−0.105			
TG	0.000	0.001	−0.072	−0.260			
ESR	−0.002	0.007	−0.049	−0.279			
CI	0.081	0.176	0.097	0.460			

Dependent variable was CD68/Cleaved GSDMD_RI. eGFR1–2 indicates CKD stages 1–2 as defined by eGFR criteria.

CD68 = cluster of differentiation 68 (macrophage marker), CI = Chronicity Index, CKD = chronic kidney disease, COD = course of disease, CRP = C-reactive protein, eGFR = estimated glomerular filtration rate, ESR = erythrocyte sedimentation rate, GSDMD = gasdermin D, ISA = immunosuppressive agents, LN = lupus nephritis, SCr = serum creatinine, SE = standard error, SLEDAI = Systemic Lupus Erythematosus Disease Activity Index, TC = total cholesterol, TG = triglyceride, UA = uric acid.

* *P* < .05.

** *P* < .01.

Supplementary note: As studies have shown that class IV LN has the highest disease activity index and the most severe inflammation, and given that macrophage pyroptosis is inherently pro-inflammatory, class IV cases were selected as the control variable in our analysis.^[19]^

## 4. Discussion

In recent years, pyroptosis has emerged as a prominent research focus, playing a crucial role in the development of various diseases. Studies have shown that pyroptosis is involved in the development and progression of renal diseases, including diabetic nephropathy, acute kidney injury, and chronic kidney disease.^[20-22]^ In a lupus mouse model (MRL/lpr), the expression of pyroptosis-related factors such as NLRP3 and caspase-1 increased with disease progression.^[23]^ Furthermore, LN patients undergoing a combination regimen of prednisone, mycophenolate mofetil, and tacrolimus have inhibited caspase-1/GSDMD-mediated pyroptosis, which delays disease progression.^[24]^ These findings underscore a significant association between pyroptosis and the development of LN. Although pyroptosis has been identified in LN, the relationship between macrophage pyroptosis and LN has not been previously explored. Our study is the first to investigate this relationship. Double immunofluorescence and ELISA results revealed significant macrophage pyroptosis in the kidney tissue of the LN group.

It is well known that the typical pathological feature of LN is the deposition of immune complexes in the kidney.^[25]^ In SLE, defective apoptosis, breakdown of self-tolerance, and impaired cellular clearance lead to systemic accumulation of autoantibodies and free nucleosomes, driving pathogenic immune complex deposition in the kidneys and subsequent structural and functional deterioration.^[26]^ Immune complexes consist of self-derived nucleic acids, their bound proteins, and cognate autoantibodies, forming pathogenic aggregates in autoimmune diseases.^[27]^ Studies suggested that immune complexes may activate the NLRP3 inflammasome, potentially triggering inflammatory responses.^[28,29]^ The pathophysiology of LN is characterized by autoantibody production. According to the EULAR/ACR-2019, the most prevalent autoantibodies include antinuclear antibodies (ANA) and anti-dsDNA antibodies, which are established biomarkers for SLE.^[30]^ Min et al demonstrated that immune complexes containing dsDNA trigger NLRP3 inflammasome-mediated IL-1β secretion in human monocytes.^[31]^ Research has confirmed that the interaction between anti-dsDNA antibodies and macrophages induces apoptotic pathways, ultimately causing renal injury.^[32]^ The LN group exhibited significantly higher anti-dsDNA antibody titers than MCD group (*P* < .001). Although the initial Spearman analysis suggested a positive correlation between antibody levels and macrophage pyroptosis, this association did not reach statistical significance in the hierarchical multiple regression analysis. This discrepancy highlights the exploratory nature of our initial univariate correlations, which did not account for multiplicity or clinical covariates, and underscores the necessity of the subsequent hierarchical multiple regression analysis. This may be attributed to the limited sample size (n = 49) and the inclusion of multiple covariates, such as immunosuppressive therapy, disease duration, and other clinical variables.

The complement system exhibits dual roles in LN pathogenesis. It facilitates the protective clearance of immune complexes (consuming C3/C4 and lowering their serum levels) while also contributing to renal injury when excessively activated by these complexes.^[33,34]^ Notably, the reduction in C3/C4 levels serves as a reliable indicator of disease activity, as corroborated by our LN cohort. This phenomenon likely stems from the overconsumption of complement components due to the deposition of immune complexes.^[35,36]^ These findings firmly establish C3/C4 as valuable diagnostic biomarkers for SLE. Emerging evidence further suggests a link between complement activation and pyroptosis.^[19,37]^ Complement inhibitors hold great promise in the treatment of LN, as they can alleviate immune injury and preserve renal function.^[38]^ However, the relationship between complement inhibitors and macrophage pyroptosis in LN remains elusive, presenting an exciting therapeutic target that warrants further investigation. This observation warrants further investigation. In our correlation analysis, we observed an inverse association between glomerular macrophage pyroptosis and complement C3/C4 levels. However, in the hierarchical multiple regression analysis, C3/C4 were not identified as significant factors associated with the level of renal macrophage pyroptosis. This discrepancy might be attributed to the limited sample size, which diminishes statistical power, as well as the inherent biological variability in complement dynamics. This discrepancy might be attributed to the limited sample size, which diminishes statistical power, as well as the inherent biological variability in complement dynamics.^[38]^ Persistent hypocomplementemia not only reflects disease activity but also predicts poorer renal outcomes. Therefore, closely monitoring complement levels in the management of LN and implementing timely therapeutic interventions are of utmost importance.

In addition to glomerular injury, tubulointerstitial damage is a prominent feature in LN, affecting approximately 50% of patients.^[39]^ The prognosis of LN is closely associated with the extent of tubulointerstitial injury, which manifests pathologically as tubular epithelial cell damage, interstitial fibrosis, and inflammatory cell infiltration.^[40]^ These deposits can activate the complement system, leading to tissue damage and inflammatory responses. These deposits can activate the complement system, leading to tissue damage and inflammatory responses.^[41]^ Emerging evidence highlights the pivotal roles played by Toll-like receptors (TLRs) and the NLRP3 inflammasome in mediating interstitial inflammation in LN.^[42,43]^ A separate study has confirmed the central role of macrophages in mediating inflammatory responses and fibrotic progression within the renal interstitium of LN.^[44]^ Luo et al identified abnormal gasdermin E (GSDME) expression in the renal tubules of both SLE patients and murine lupus models, suggesting the potential involvement of GSDME-mediated pyroptosis in the disease pathogenesis.^[45]^ Notably, substantial expression of the pyroptosis executioner protein GSDMD was observed in both tubular and interstitial cells from LN patients and experimental lupus mice, providing novel mechanistic insights into LN-associated tubulointerstitial injury. 24 Our study identified the presence of macrophage pyroptosis in the renal interstitium of LN patients, although the precise mechanisms require further investigation. Tubulointerstitial inflammation and its severity are also associated with the risk of renal failure in LN.^[46]^ In clinical practice, CRP is regarded as a nonspecific marker of persistent inflammation. Elevated CRP levels in LN patients are significantly correlated with interstitial inflammatory cell infiltration, tubulointerstitial atrophy, and interstitial fibrosis.^[39]^ Our study demonstrated that serum CRP levels were significantly higher in LN patients compared to controls (*P* < .05). Initial Spearman correlation analysis revealed a positive association between CRP levels and the extent of interstitial macrophage pyroptosis (*R* = 0.412, *P* = .003). In the hierarchical multiple regression analysis, CRP ≥ 5 was significantly associated with interstitial macrophage pyroptosis after adjusting for potential confounders. However, this association was no longer statistically significant when the analysis was stratified by immunosuppressive therapy subgroups. This discrepancy may be attributed to the complex regulation of CRP expression, which involves genetic polymorphisms (e.g., CRP rs1205) and the activation status of the type I interferon signature, as well as the relatively small sample size of both the overall cohort and the subgroup of patients not receiving immunosuppressive therapy.^[47,48]^ Our study identified a significant positive correlation between ESR, another inflammatory marker, and the degree of interstitial macrophage pyroptosis (β = 0.298, *P* = .018). Previous studies have shown that ESR, as an indicator of systemic autoimmune disease activity, is significantly associated with SLE disease activity.^[49]^ Nevertheless, given the low specificity of ESR, which can be influenced by anemia, infections, and malignancies, larger-scale studies are needed to validate these findings. Clinically, elevated ESR in LN patients necessitates a comprehensive evaluation of both disease activity and potential infection risks to optimize disease management. Previous studies have demonstrated that eGFR is closely associated with both disease activity and inflammatory response in LN.^[50]^ We found that eGFR levels were significantly lower in LN patients compared to control subjects (*P* < .001), which is consistent with previous findings. Notably, Xie’s research team revealed that podocyte pyroptosis and its associated inflammatory mediators contribute to renal cell injury, leading to impaired glomerular filtration and accelerated LN progression.^[51]^ These findings provide a mechanistic basis for developing therapeutic strategies targeting pyroptosis pathways to preserve renal function. Further stratified regression analyses were performed after adjusting for age, sex, and other potential confounders. Based on the eGFR staging criteria, LN patients were categorized into 3 groups: CKD stages 1–2, stages 3–4, and stage 5. Our hierarchical multiple regression analysis revealed that, after adjusting for potential confounders, both CKD stages 3–4 and stage 5 were independently associated with higher levels of interstitial macrophage pyroptosis, with the most pronounced difference observed in CKD stage 5. This finding suggests a cross-sectional association between advanced CKD stage and elevated macrophage pyroptosis levels. From a clinical perspective, this observation raises the hypothesis that patients with advanced LN might potentially benefit from therapies targeting pyroptosis. However, given the cross-sectional nature of our study, these therapeutic implications remain speculative and require validation through large-scale clinical trials.

Huang et al demonstrated NLRP3 expression in tubular cells of class IV LN patients, with NLRP3 activation showing positive correlation with disease activity index scores.^[52]^ A study revealed a significant association between circulating caspase-1/PI-positive cell counts and SLEDAI. 24 However, the clinical predictive value of caspase-1/PI double-positive cells for disease activity and treatment response requires further validation. Notably, in our current study, no significant correlation was observed between SLEDAI and macrophage pyroptosis. This discrepancy may stem from the fact that SLEDAI primarily focuses on clinical outcomes and systemic manifestations, incorporating various extrarenal symptoms rather than specific renal pathological processes.

Our study also showed that LN patients had low vitamin D (Vit D) levels, which is consistent with the findings of Abediazar et al.^[53]^ In vitro research has shown that Vit D exerts a kidney-protective effect against LN autoantibodies: serum 25(OH)2D3 is associated with reduced aberrant autophagy in podocytes.^[54]^ Vit D has been shown to prevent kidney tissue damage, particularly in the renal interstitium, by decreasing pyroptosis.^[55]^ Our study found a negative correlation between Vit D levels and renal interstitial macrophage pyroptosis. Hierarchical multiple regression analysis revealed a significant association between Vit D levels and renal interstitial macrophage pyroptosis. Notably, when the analysis was stratified to include only patients not receiving immunosuppressive therapy, this association was substantially attenuated and did not reach statistical significance. Although this association was not statistically significant in the final model, close attention to Vit D status in LN patients may still be warranted, and appropriate supplementation strategies could be considered as part of comprehensive clinical management.

Our study showed that the expression levels of IL-1β and IL-18 were significantly elevated in both LN and MCD patients, which is consistent with previous findings by Mende and Zhuang et al.^[56,57]^ Mechanistically, as key downstream effectors of pyroptosis, IL-1β and IL-18 are released into the extracellular space upon pyroptosis activation, triggering inflammatory responses.^[10]^ In LN, these pro-inflammatory cytokines directly damage glomerular podocytes, contributing to renal injury.^[58]^ Given this mechanism, targeting the IL-1β/IL-18 pathway holds therapeutic potential. IL-1 receptor antagonists and IL-1β monoclonal antibodies have shown efficacy in inflammatory diseases such as rheumatoid arthritis and gouty arthritis. However, large-scale clinical trials in LN are still lacking, and further research is needed to evaluate their clinical translation in LN.^[59,60]^

GSDMD, a key executor of pyroptosis, plays a pivotal role in mediating inflammatory cell death. Our study observed prominent GSDMD deposition in both the glomeruli and renal interstitium of LN patients, which is consistent with the findings reported by Cao et al.^[24]^ These results suggest that dysregulated GSDMD activation may contribute to aberrant cell death in SLE and other autoimmune diseases. Notably, preclinical evidence indicates that disulfiram, a potential GSDMD inhibitor, alleviates lupus-like symptoms in PIL mice by suppressing pyroptosis.^[57]^ Given its central role in inflammatory pathogenesis, targeting GSDMD represents a promising therapeutic strategy, warranting further clinical investigation to evaluate its efficacy in autoimmune disorders.

As a single-center cross-sectional study, the current research has several inherent limitations. First, the relatively small sample size may compromise statistical power, necessitating cautious interpretation of the findings. Second, the exclusive recruitment of participants from a single regional medical center introduces potential selection bias, thereby limiting the generalizability of our conclusions. Third, given the well-established genetic predisposition of lupus nephritis, the lack of incorporation of genetic data (e.g., human leukocyte antigen haplotypes and susceptibility gene polymorphisms) may constrain comprehensive evaluation of disease heterogeneity. Fourth, inherent differences in sex distribution and treatment regimens between the MCD and LN groups may have introduced potential confounding effects, despite statistical adjustments in the regression analyses. Fifth, detailed data on immunosuppressive therapy, including specific drug doses, initiation time, and duration, were not available due to the cross-sectional design. Although we adjusted for immunosuppressive therapy as a binary covariate and performed subgroup analyses in patients not receiving treatment, residual confounding related to treatment intensity and duration cannot be entirely excluded. Finally, while healthy renal tissue would theoretically serve as the optimal reference, we utilized MCD samples as controls based on medical ethics and feasibility considerations. This approach carries potential confounding limitations, which we aim to address in subsequent in vivo experiments. To address these limitations, future studies should consider: implementing multicenter, large-scale cohort designs; enrolling ethnically and geographically diverse populations; and integrating genomic profiling data. Such methodological refinements would not only validate our observations but also enhance the clinical translatability of the findings.

## 5. Conclusions

In summary, this study is the first to describe the presence of macrophage pyroptosis within the renal tissues of patients with LN. We further identified significant correlations between the extent of macrophage pyroptosis and key clinical parameters in LN. As these findings are derived from a relatively small sample size, future multicenter studies with larger cohorts are warranted. Notably, while our investigation focused specifically on LN, macrophage infiltration constitutes a common pathological feature across other renal diseases, including diabetic nephropathy, IgA nephropathy, and hyperuricemic nephropathy. Comparative studies across these nephropathies may provide broader insights into disease-specific pyroptosis patterns. The current work primarily demonstrates an association between macrophage pyroptosis in LN and its clinical correlates. Ongoing investigations utilizing animal models and in vitro systems are actively exploring the underlying mechanisms.

## Acknowledgments

We thank all members of the Research Center of Medical Sciences (Guangxi Academy of Medical Sciences & The People’s Hospital of Guangxi Zhuang) for providing technique support for this study.

## Author contributions

**Conceptualization:** Kun Ye, Lingzhang Meng.

**Data curation:** Lingzhang Meng.

**Formal analysis:** Qian Yang, Lingzhang Meng.

**Funding acquisition:** Kun Ye.

**Investigation:** Yunfeng Huang.

**Methodology:** Qian Yang, Yunfeng Huang.

**Project administration:** Lingzhang Meng.

**Resources:** Yiyun Huang.

**Software:** Tingting Jiang, Yiyun Huang.

**Supervision:** Lingzhang Meng.

**Validation:** Qian Yang, Tingting Jiang, Lingzhang Meng.

**Visualization:** Qian Yang, Runhang Chen, Jiao Lan, Lingzhang Meng.

**Writing – original draft:** Kun Ye.

**Writing – review & editing:** Runhang Chen, Jiao Lan.
